# The Interpretation of Arterial Blood Gas During the Apneic Phase of a Patient With Obstructive Sleep Apnea: A Case Report

**DOI:** 10.7759/cureus.39184

**Published:** 2023-05-18

**Authors:** Ayaskant Sahoo

**Affiliations:** 1 Anesthesiology, Manipal Tata Medical College, Jamshedpur, IND

**Keywords:** sleep apnea monitoring, pulmonary medicine-sleep apnea-critical care medicine-insomnia, apnea phase of osa, osa in icu, abg in obstructive apnea, obstructive sleep apnea

## Abstract

Obstructive sleep apnea (OSA) is now increasingly recognized as a disease entity that can play a major role in affecting multiple organ systems. Even though the symptoms of OSA were first described in the 19th century as Pickwickian syndrome, there are a lot of things that came to be known only recently including its pathophysiology and diagnosis. In this case report, we present some findings that mostly have not been reported in OSA patients before. It has been reported that OSA patients have a typical arterial blood gas (ABG) picture of raised bicarbonate (HCO_3_-) levels, which also aid in adding to the diagnosis, but we found some more findings that are only specific to the apneic phase of the disease. A 65-year-old female patient was put on a ventilator due to dengue-associated acute respiratory distress syndrome (ARDS). She was also diagnosed with obstructive sleep apnea after facing difficulty in weaning from a ventilator. Post extubation, she was put on noninvasive ventilation (NIV), but the patient's arterial blood gas (ABG) drawn during the apneic phase was showing signs of severe metabolic acidosis even on NIV. This was reversible and gets corrected once the patient is awakened or put on NIV. Clinical decisions from ABG in a patient with OSA may result in errors especially when the ABG is drawn during the apneic phase of the disease. Clinicians have to be careful of this phenomenon, and more research needs to be undertaken to fully understand the pathophysiology of this phenomenon.

## Introduction

Obesity is common in patients admitted to the ICU, but obstructive sleep apnea (OSA) is not studied in detail in the ICU setup. Most of the articles published are related to the complications surrounding the surgery and anesthesia during bariatric surgeries or in the postoperative period. The prevalence of OSA is between 5% and 24%, and in the ICU, the prevalence of documented OSA was estimated to be around 8% [1]. Even though bicarbonate (HCO_3_-) levels are recognized to be a screening tool for OSA [2], we could hardly find any literature that documents bicarbonate levels during the apneic phase of OSA. Here, we present a case of a 65-year-old female who was admitted to the ICU for dengue-associated acute respiratory distress syndrome (ARDS). In the course of the disease, she was intubated and put on mechanical ventilation. She made a good recovery from dengue-associated ARDS, but the presence of a pre-existing non-documented obstructive sleep apnea brought about a tricky situation to handle during the phase of weaning from a ventilator.

## Case presentation

A 65-year-old patient was admitted post dengue fever and was diagnosed with dengue-associated ARDS needing ICU management. The patient made a good recovery, and after a successful weaning trial, she was extubated. After five hours of extubation from a ventilator, the patient developed a hypoxic spell with oxygen saturation (SpO_2_) dropping to mid-70s. Oxygen was supplemented, and arterial blood gas (ABG) was sent immediately. Arterial blood gas (ABG) analysis showed severe metabolic acidosis (pH of 6.813) and bicarbonate of 8.3 mmol/L falling from 34.5 mmol/L from the previous reading. In the first two instances of apneic spell, the acidosis was corrected by giving bicarbonate as the pH was below 7.1. She was put on intermittent noninvasive ventilation (NIV), and over the next two days, it was observed that the patient was getting apneic spells after sleeping, and pH changes were significant during this phase. Though partial pressure of carbon dioxide (PaCO_2_) changes were not significant, bicarbonate fall was significant during the apneic spells. Partial pressure of oxygen (PaO_2_) levels during apneic spells as per our ABG samples seem normal because oxygen was supplemented before drawing the ABG samples, but pH and HCO_3_- take time to normalize after the patient wakes up (Table 1).

**Table 1:** ABG values in chronological order Extreme pH and corresponding HCO_3_- values are mentioned in bold ABG, arterial blood gas; HCO_3_-, bicarbonate; PaCO_2_, partial pressure of carbon dioxide; AnGap, anion gap

Day, time	pH	PaCO_2_ (mm Hg)	PaO_2_ (mm Hg)	HCO_3_- (mmol/L)	AnGap	Na+ (mmol/L)	Cl- (mmol/L)
Day 1, 3 pm	7.376	56.1	103.7	32.2	7.3	137	102
Day 1, 6 pm	7.366	61.6	62.9	34.5	5.1	138	102
Day 1, 10 pm	6.813	52.7	122.1	8.3	18.1	129	106
Day 1, 11:07 pm	7.087	50.1	132.7	14.7	-16.5	116	121
Day 1, 11:09 pm	6.894	52.4	132.1	9.9	10	122	106
Day 1, 11:12 pm	6.922	46.2	135.6	9.3	3.7	117	108
Day 2, 7 am	7.372	56.6	122.3	32.1	16.1	132	87
Day 2, 10:53 am	6.736	52.2	134.2	6.9	13.4	126	109
Day 2, 12 pm	7.394	59.7	115.7	35.7	6.1	137	100
Day 2, 2:30 pm	7.426	52.2	122.9	33.5	10.7	133	93
Day 2, 8 pm	7.393	63.8	104.9	38	5.2	137	98
Day 3, 7 am	7.359	70	89.6	38.6	5.3	136	97
Day 3, 7:30 pm	7.472	46.6	47.2	33.3	12.7	131	89
Day 4, 12 pm	7.445	53.1	61.6	36.5	-1	128	92

In the first two instances of metabolic acidosis, we had to correct the bicarbonate levels to correct the extremely low pH but only once we had a suspicion of OSA in our patient. The use of STOP-BANG questionnaire-based and overnight pulse oximetry-based [3] screening methods helped us to come to a conclusion of OSA, which was complicating the situation. Following the diagnosis, with every sudden drop in SpO_2_, our patient was connected to noninvasive ventilation and slowly weaned off. This was continued until her general condition improved in the ICU. Before discharge, the family members were counselled for polysomnography, which was done, and it confirmed our diagnosis of OSA.

## Discussion

With this report, we want to highlight that there are very limited studies on arterial blood gas levels in OSA patients that are admitted to the ICU for diseases apart from bariatric surgeries. Even though done for diagnosis purposes, ABG analysis done especially during the apneic phase is extremely rare in the available literature. Our patient was showing severe metabolic acidosis during apneic spells and near-typical ABG values of an OSA patient. Once a clinical suspicion is raised in the ICU, a questionnaire-based screening tool may be used to make a provisional diagnosis, i.e., STOP-BANG [4], Epworth Sleepiness Scale (ESS) [5], and Berlin questionnaire [6]. Out of the three tools, a meta-analysis [7] found the sensitivity of STOP-BANG [4] questionnaire to be superior to the rest. In their study, Chung et al. [2] proposed adding serum bicarbonate levels to the STOP-BANG questionnaire to improve the specificity and prediction of moderate to severe OSA. In their paper, Spoletini et al. [8] proposed using bicarbonate levels as a screening tool for sleep-disordered breathing in obese patients. Our patient indeed had bicarbonate levels above 30 mmol/L in most ABG reports except those taken during the apneic spells, and the PaCO_2_ levels were also persistently above 50 mm Hg in most ABGs; the two findings are consistent with OSA patients. According to the PaCO_2_ levels, we can classify our patient into a moderately severe OSA category [9]. In their study, Bailly et al. [10] showed that patients with OSA without associated diseases did not have a significant impact on ICU stay, but patients with OSA and morbid obesity did have a longer stay in the ICU, whereas obstructive sleep apnea was associated with a reduction in both ICU and hospital mortality as per the paper published by Bolona et al. [1].

## Conclusions

With this case report, we want to emphasize that pre-existing, undiagnosed OSA can be very tricky in an ICU setting especially during weaning from a ventilator. We propose that more research may be taken up in this regard to find out any missing link especially during the apneic phase of OSA patients. Studies must be conducted to standardize the ABG values especially during the apneic phase of OSA patients. The apneic phase, as noticed in our patient, may lead to severe metabolic acidosis, which can be managed without alkali correction by simply putting the patient on continuous positive airway pressure (CPAP) or NIV therapy for a short duration.

Polysomnography cannot be done in most patients in resource-limited ICU setups, and hence, adequate awareness and the use of the screening methods of OSA can be used to successfully diagnose and manage such patients.
